# Polyglutamine Genes Interact to Modulate the Severity and Progression of Neurodegeneration in *Drosophila*

**DOI:** 10.1371/journal.pbio.0060029

**Published:** 2008-02-12

**Authors:** Derek Lessing, Nancy M Bonini

**Affiliations:** Department of Biology, University of Pennsylvania, Howard Hughes Medical Institute, Philadelphia, Pennsylvania, United States of America; Baylor College of Medicine, United States of America

## Abstract

The expansion of polyglutamine tracts in a variety of proteins causes devastating, dominantly inherited neurodegenerative diseases, including six forms of spinal cerebellar ataxia (SCA). Although a polyglutamine expansion encoded in a single allele of each of the responsible genes is sufficient for the onset of each disease, clinical observations suggest that interactions between these genes may affect disease progression. In a screen for modifiers of neurodegeneration due to SCA3 in *Drosophila*, we isolated *atx2*, the fly ortholog of the human gene that causes a related ataxia, SCA2. We show that the normal activity of Ataxin-2 (Atx2) is critical for SCA3 degeneration and that Atx2 activity hastens the onset of nuclear inclusions associated with SCA3. These activities depend on a conserved protein interaction domain of Atx2, the PAM2 motif, which mediates binding of cytoplasmic poly(A)-binding protein (PABP). We show here that PABP also influences SCA3-associated neurodegeneration. These studies indicate that the toxicity of one polyglutamine disease protein can be dramatically modulated by the normal activity of another. We propose that functional links between these genes are critical to disease severity and progression, such that therapeutics for one disease may be applicable to others.

## Author Summary

Six forms of spinal cerebellar ataxia (SCA1, −2, −3, −6, −7, and −17) are caused by dominant mutations in the respective genes. Patients suffering from these different forms of SCA have similar symptoms of progressive adult-onset neurodegeneration, and the same causative mutation, a CAG repeat expansion encoding an expanded run of polyglutamine. Although the affected proteins are distinct and share no sequence similarity beyond the polyglutamine domains, clinical and other observations hint at interactions between the genes that cause different forms of SCA. Using *Drosophila* as a model for human disease, we now detail an interaction between the genes associated with SCA3 and SCA2. We find that toxicity and neurodegeneration induced by pathogenic forms of SCA3 depend on the normal activity of the fly counterpart of the gene associated with SCA2, *ataxin-2*. This interaction depends on a conserved protein-interaction motif of Ataxin-2, and a protein that binds this motif, cytoplasmic poly(A)-binding protein (PABP), also modulates SCA3 degeneration. These results suggest that the normal roles of Ataxin-2 and PABP, potentially to regulate the translation of select target mRNAs, are critical to SCA3 disease. These studies also highlight how a fly model can serve to enhance and extend intriguing clinical findings of the human disease.

## Abstract 3

In a fly model of spinal cerebellar ataxia type 3 (SCA3), toxicity of the pathogenic human protein is shown to depend on the normal activity of the fly ortholog of a gene that causes the related human disease, SCA2.

## Introduction

The polyglutamine diseases are caused by the expansion of a CAG repeat encoding glutamine within the open reading frames of at least nine genes [1]. The disease state is thought to be due to a misfolded conformation of the pathogenic protein (termed an Ataxin in most forms of spinal cerebellar ataxia [SCAs]), which accumulates intracellularly in ubiquitinated inclusions along with chaperones and subunits of the proteasome [2]. Among the SCAs, there is a remarkable overlap of symptoms, including progressive loss of gait and limb muscle coordination, with neurodegeneration in the cerebellum and often select brainstem nuclei [3].

In addition to the shared mechanism of mutation and clinical phenotypes, there are intriguing hints of interactions between the polyglutamine genes that cause different SCAs. For example, relatively long CAG repeat lengths (still within the normal range) in the causative gene for SCA6 are correlated with an early age of onset of SCA2 [4]. In addition, there are a number of such observations suggesting links between SCA2 and SCA3 diseases. The severity of facial muscle twitch in patients with SCA3 is correlated with the length of the CAG repeat in the normal alleles of the SCA2 gene *ATXN2* [5]. Both SCA2 and SCA3 are unusual among the dominantly inherited ataxias in that they can manifest with parkinsonism [6]. Finally, the normal Ataxin-2 (Atx2) protein can be detected in the pathogenic inclusions of SCA3 patients, and, likewise, normal Atx3 protein localizes to the inclusions formed in SCA2 patients [7].

Several SCAs have been successfully modeled in *Drosophila* [8–10], an organism ideally suited to screen for genes that can modify the effects of protein toxicity and neurodegeneration. Moreover, up-regulation of *atx2*, the *Drosophila* ortholog of the human gene that causes SCA2 disease, has been shown to enhance the toxicity of human disease forms of SCA1 and SCA3 in flies [11]. Many genes that modify SCA1 toxicity in the fly, including *atx2*, have been shown to encode proteins that interact directly with pathogenic Atx1, and further, these proteins form an interaction network that includes Atx3 [12]. Taken together with the clinical data, this work suggests that interactions between CAG-containing genes may occur that influence disease.

Despite these findings, the molecular activities of the Atx2 and Atx3 proteins do not indicate obvious functional links between these two particular CAG repeat genes. Atx2 is a cytoplasmic protein with an RNA-binding Lsm domain and a PAM2 motif that binds specifically to the PABC domain of proteins such as the cytoplasmic poly(A)-binding protein (PABP) [13,14]. PABP and Atx2 directly interact in Caenorhabditis elegans [15]; and in flies and cultured cells, PABP and Atx2 also physically associate and co-sediment with polyribosomes [16]. In contrast, Atx3 shuttles between the cytoplasm and nucleus, contains ubiquitin interaction motifs and de-ubiquitination activity [17–20], and is normally neuroprotective, an activity that depends on a fully functional proteasome [21].

In a screen in *Drosophila* for modifiers of degeneration induced by the human pathogenic protein causing SCA3, we identified *atx2*. Up-regulation of *atx2* synergistically enhanced SCA3 degeneration, and strikingly, we found that the endogenous activity of *atx2* modulates progression of neurodegeneration induced by pathogenic Atx3. Thus, toxicity of the polyglutamine disease protein Atx3 is critically dependent upon the normal activity of *atx2*, a second gene associated with polyglutamine disease in humans. These findings underscore the power of the *Drosophila* system, and provide a foundation for further molecular insight into human genetic and clinical studies.

## Results

### Up-Regulation of Atx2 Synergistically Enhances Atx3-Induced Neurodegeneration

**Figure 1:**
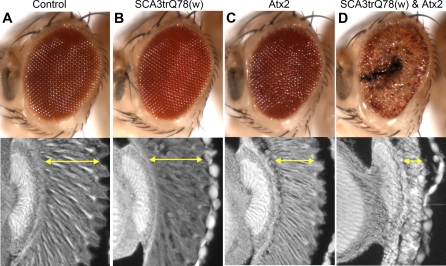
Atx2 Enhances Atx3-Dependent Neurodegeneration External eye (top) and internal retina sections (bottom) of 1-d-old males. Internal degeneration is reflected in the thickness of the retina, indicated by the yellow double-headed arrows. (A) Control fly with driver only; eye is normal with a highly regular structure and normal pigmentation. Genotype: *Gmr-Gal4/+*. (B) Flies with a weakly expressing insertion of SCA3trQ78 have a normal external eye, although internally the retina is disorganized (compare to panel [A]). Genotype: *Gmr-Gal4/+; UAS-SCA3trQ78(w)/+*. (C) Flies expressing Atx2 have a mildly rough external eye surface, but no obvious degeneration externally or internally. Genotype: *Gmr-Gal4/UAS-Atx2*. (D) Expression of Atx2 with SCA3trQ78 results in severe degeneration, with loss of pigmentation externally and collapse of the retina internally. Genotype: *Gmr-Gal4/UAS-Atx2; UAS-SCA3trQ78(w)/+*.

Expression of pathogenic human Atx3 from the transgene *UAS-SCA3trQ78* in the developing eye under the control of the *Gmr-Gal4* driver causes degeneration [8]. We identified a transposon insertion in *atx2* conferring Gal4-dependent expression that greatly enhanced this toxicity (also reported in [11]; see Materials and Methods for details of the screen). To verify that up-regulation of *atx2* was responsible for the effect, we coexpressed a *UAS-Atx2* transgene [22] together with pathogenic Atx3 and confirmed strongly enhanced eye degeneration with loss of pigmentation and severe collapse of the retina (Figure 1). Coexpression of Atx2 with a nonpathogenic Atx3 construct bearing a normal-length polyglutamine repeat had no effect, such that the eyes looked the same as those expressing only Atx2, as in Figure 1C (unpublished data).

**Figure 2:**
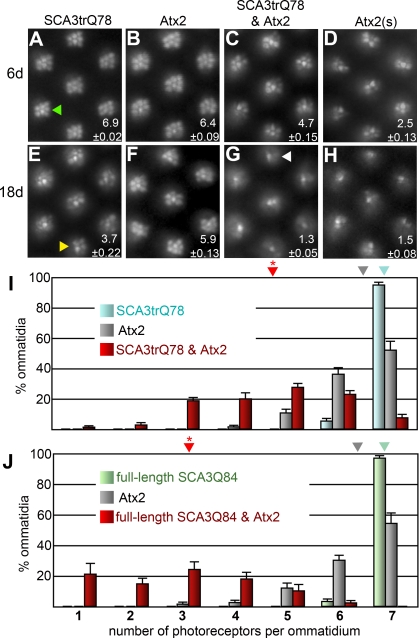
Adult-Onset Degeneration due to Pathogenic Atx3 Is Synergistically Enhanced by Atx2 (A–H) Pseudopupil preparations: each panel shows a field of seven unit eyes (ommatidia); each ommatidium has seven visible photoreceptors (PR) with light-gathering organelles (rhabdomeres) in a characteristic trapezoidal pattern (e.g., green arrowhead in [A]). Examples of degenerate ommatidia are highlighted in (E) by a yellow arrowhead (4 PR) and in (G) by a white arrowhead (1 PR). The mean number of PR ± SEM per ommatidium (*n* = 10 flies) is indicated at the bottom right of each panel. (A–D) shows 6-d-old adults; (E–H) 18-d-old adults. (A and E) Expression of SCA3trQ78 causes adult-onset PR loss over 18 d (from a mean of 7.0 at 1 d to 3.7). Genotype *UAS-SCA3trQ78(s)/+; Rh1-Gal4/+*. (B and F) Mild degeneration occurs with up-regulation of Atx2, to a mean of 5.9 PR at 18 d. Genotype *UAS-Atx2/+; Rh1-Gal4/+*. (C and G) Coexpression of SCA3trQ78 with Atx2 causes severe PR loss (to a mean of 4.7 as early as 6 d and to 1.3 at 18 d). Genotype *UAS-Atx2/UAS-SCA3trQ78(s); Rh1-Gal4/+*. (D and H) Strong expression of Atx2 alone causes severe degeneration (to a mean of 1.5 PR at 18 d). Genotype: *UAS-Atx2(s)/+; Rh1-Gal4/+*. (I) Distribution of ommatidia in 6-d flies. The mean numbers of PR are indicated schematically by the positions of arrowheads at top relative to the *x*-axis (* *p* < 0.001 vs. SCA3trQ78 alone, *p* < 0.05 vs. Atx2 alone). Genotypes as in (A–C), and mean PR counts are listed in lower right corners of (A–C). (J) The full-length pathogenic Atx3 protein also interacts synergistically with Atx2: distribution of PR at 18–23 d; see text for mean PR counts, also indicated by triangles at the top relative to the *x*-axis (* *p* < 0.001 vs. SCA3Q84 and *p* < 0.05 vs. Atx2). Genotypes: (green) *UAS-SCA3Q84/+; Rh1-Gal4/+*, (grey) *UAS-Atx2/+; Rh1-Gal4/+*, and (red) *UAS-Atx2/UAS-SCA3Q84; Rh1-Gal4/+*.

Since Atx2 expression can cause developmental effects [22], we further examined neuronal integrity in an adult-onset situation using the driver *Rhodopsin-1-Gal4* (*Rh1-Gal4*), active selectively in differentiated photoreceptor neurons (PR). Adult flies expressing pathogenic Atx3, normal fly Atx2, or both were born with the normal complement of seven visible PR per unit eye, or ommatidium, as seen in pseudopupil preparations (see Materials and Methods). Strong expression of SCA3trQ78 induced progressive degeneration, from seven PR declining to 3.7 ± 0.22 PR by 18 d (Figure 2A and 2E). Flies expressing only Atx2 also underwent retinal degeneration, but to a lesser extent (5.9 ± 0.13 PR at 18 d; Figure 2B and 2F; and see below). Coexpression of Atx2 with Atx3, however, resulted in dramatically enhanced degeneration, such that even by 6 d, there were only 4.7 ± 0.15 PR (Figure 2C) compared to the near-normal eyes resulting from expression of either protein alone (Figure 2A, 2B, and 2I). By 18 d, these flies had undergone nearly complete PR loss (Figure 2G). At either 6 d or 18 d, PR loss due to coexpression was greater than the sum of the deficits caused by each transgene alone, indicating a synergistic interaction.

These experiments were performed with a truncated version of pathogenic Atx3 [8]. In mouse models and in human SCA3 disease, the full-length pathogenic protein undergoes proteolysis, yielding a similarly sized fragment containing the polyglutamine tract that is thought to contribute to disease [23]. To determine whether Atx2 interacted with the intact as well as truncated Atx3 protein, we coexpressed Atx2 with full-length pathogenic Atx3 (SCA3Q84 [21]). Under conditions where Atx2 or SCA3Q84 alone had mild effects (6.3 ± 0.16 PR and 6.97 ± 0.02 PR, respectively), coexpression of Atx2 with SCA3Q84 resulted in strikingly severe degeneration (2.9 ± 0.30 PR; Figure 2J). Thus, synergistic enhancement of degeneration by Atx2 was not limited to truncated versions of pathogenic Atx3, but extended to the full-length protein. To further determine the specificity of Atx2, we coexpressed Atx2 with two different Huntington disease transgenes [24,25]. Atx2 dramatically enhanced toxicity of an exon-1 Huntingtin (Htt) protein, but had no effect on toxicity of a longer Htt fragment (Figure S1). Cleavage of the Huntingtin protein contributes to disease [26,27]; Atx2 may play a role by selectively promoting toxicity of small Htt fragments.

For some dominantly acting disease proteins, up-regulation of the normal version of the protein is sufficient to cause neurodegeneration clinically (for example, [28]) and in animal models [9,29,30]. We were therefore interested in the extent to which normal Atx2 could induce degeneration in the adult in the absence of developmental effects. The modest photoreceptor loss due to expression of Atx2 seen in Figure 2B and 2F was more striking with stronger expression of Atx2 from the transgenic line *UAS-Atx2(s)* [22]; even 1-d flies had mild but significant loss of PR neurons (6.8 ± 0.06, vs. 7.0 ± 0.01 PR in flies expressing SCA3trQ78 only; *p* = 0.009) that progressed rapidly to 1.5 ± 0.08 PR by 18 d (Figure 2D and 2H). Therefore, up-regulation of normal Atx2 without a polyglutamine expansion causes severe, progressive degeneration.

### Reduction of Atx2 Activity Mitigates SCA3 Degeneration

**Figure 3:**
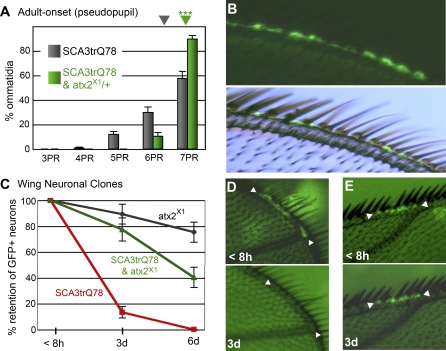
Atx2 Function Is Required for Atx3-Induced Neurodegeneration (A) Heterozygous loss of Atx2 suppresses SCA3trQ78 degeneration in the adult-onset model (*** *p* = 0.0007). Distribution of ommatidia in 12-d flies with either normal Atx2 function or with heterozygous loss of Atx2 activity. Grey bars: *UAS-SCA3trQ78(s)/+; Rh1-Gal4/+*. Green bars: *UAS-SCA3trQ78(s)/+; Rh1-Gal4/atx2^X1^*. (B) Clones of sensory neurons in the anterior wing margin labeled with GFP by the MARCM technique [32]. Top, membrane-bound GFP marks neurons expressing *UAS-SCA3trQ78(s)*; bottom, GFP signal merged with bright-field image. (C) Loss of Atx2 function in neuronal clones homozygous for *atx2^−^* protects neurons from SCA3trQ78 toxicity. The retention of GFP^+^ neurons present in newly born adults is plotted over time (mean ± SEM, *n* = 10 flies per genotype). Whereas SCA3trQ78 expression alone results in rapid loss of neurons (red), expression of SCA3trQ78 together with homozygous loss of *atx2* results in significantly delayed neuron loss (green). Loss of *atx2* alone has a minimal effect (black). (D and E) Images of two wings, at two time points each (<8 h and 3 d). Arrowheads flank GFP^+^ neurons in the anterior margin in young animals. (D) Wing from a fly expressing SCA3trQ78 in GFP^+^ neurons with normal Atx2 activity. Neurons visible when the fly was less than 8 h old (top) are no longer detectable at 3 d (bottom). (E) Wing from a fly expressing SCA3trQ78 in GFP^+^ neurons that also lack all Atx2 activity (homozygous for a null allele, *atx2* ^X1^/*atx2* ^X1^); the GFP signal is maintained over 3 d. See Materials and Methods for genotypes for (B–E). Proximal is to the left in (B, D, and E).

Our findings demonstrated that up-regulation of Atx2 activity modulates SCA3 pathogenesis. To determine whether Atx2 normally contributes to disease, we examined the effects of reducing endogenous Atx2, which is expressed abundantly in the adult fly and is enriched in the head and brain [31]. Flies lacking 50% of normal Atx2 (heterozygous for a null *atx2* allele) were examined for modulated SCA3trQ78 toxicity in pseudopupil preparations with adult-onset expression. At 12 d, when Atx3 normally caused photoreceptor loss to 6.4 ± 0.08 PR, reduction of Atx2 by 50% significantly mitigated degeneration (6.9 ± 0.03 PR, Figure 3A). This finding was confirmed with a deletion removing *atx2* (unpublished data). Similar studies showed that flies expressing full-length pathogenic Atx3 also showed mitigated PR loss when heterozygous for an *atx2* deletion (Figure S2).

To further address the role of endogenous *atx2*, we analyzed neuronal integrity using the MARCM method [32], whereby we could analyze small clones of neurons lacking *atx2* activity. Neuronal clones selectively marked with green fluorescent protein (GFP) (Figure 3B) were induced by mitotic recombination and were either *atx2^+^* or homozygous mutant for *atx2*. When the *UAS-SCA3trQ78* transgene was present, it was expressed only in neuronal clones along with the GFP marker. GFP^+^ sensory neurons in the anterior wing margin were examined in young adult flies less than 8 h old and over the subsequent 6 d, following marked neurons in individual animals. In the absence of pathogenic Atx3, GFP^+^ neurons that were *atx2* ^+^ or homozygous for a null allele of *atx2* were retained at a similar rate: 83% of the former (unpublished data) and 75% of the latter (Figure 3C) remained at 6 d. In contrast, of GFP^+^ neurons expressing SCA3trQ78 that were present in young animals, only 13% were still detectable at 3 d, with all neurons lost by 6 d (Figure 3C and 3D). Strikingly, SCA3trQ78-expressing neurons were dramatically retained if they also lacked Atx2 function: 77% of these neurons were present at 3 d and 40% were retained at 6 d (Figure 3C and 3E). Taken together, these studies indicate that normal endogenous Atx2 activity facilitates Atx3-dependent toxicity, with up-regulation of Atx2 enhancing and loss of Atx2 function dramatically slowing the progression of neurodegeneration caused by pathogenic Atx3.

### Up-Regulation of Atx2 Accelerates Inclusion Formation by Pathogenic Atx3

**Figure 4:**
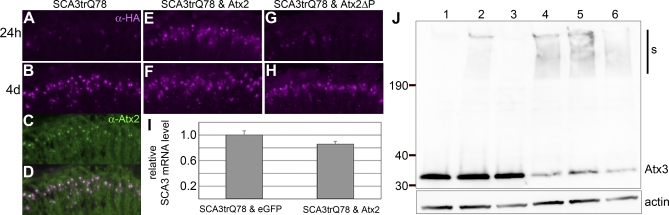
Atx2 Alters the Time Course of and Colocalizes with Pathogenic Atx3 Inclusions Retinal cryosections at (A, E, and G) 24 h and (B–D, F, and H) 4 d. (A–D) Flies expressing SCA3trQ78 only (genotype *UAS-SCA3trQ78(s)/+; Rh1-Gal4/+*). (E and F) Flies coexpressing SCA3trQ78 and Atx2 (*UAS-SCA3trQ78(s)/UAS-Atx2; Rh1-Gal4/+*). (G and H) Flies coexpressing SCA3trQ78 and Atx2ΔP (*UAS-SCA3trQ78(s)/+; Rh1-Gal4/UAS-Atx2ΔP*). (A, B, and E–H) Pathogenic Atx3 protein is tagged with the HA epitope and visualized with anti-HA (magenta). Up-regulation of Atx2, but not of Atx2ΔP lacking the PAM2 motif, results in the early onset of inclusions at 24 h. (C) Same section as in (B), stained for endogenous Atx2 (green). (D) Merged images show colocalization of Atx2 in the inclusions. See Figure S3 for larger images, including DAPI staining, that show nuclear localization and orientation within the retina. (I) Coexpression of SCA3trQ78 with Atx2 does not significantly change SCA3 mRNA levels (*p* = 0.21). Quantitative real-time PCR comparing mean relative levels ± SEM of SCA3trQ78 mRNA from heads of 1–2-d flies, from two separate cDNA preparations for each group, normalized to coexpression with control transgene *UAS-eGFP*. Genotypes: *UAS-SCA3trQ78(s)/UAS-eGFP*; *Rh1-Gal4/+*, and *UAS-SCA3trQ78(s)/UAS-Atx2; Rh1-Gal4/+*. (J) Immunoblot of the Atx3 protein (anti-HA, top) and loading control β-actin (bottom). Lanes 1–3, head extracts from 24-h flies; lanes 4–6, heads extracts from 4-d flies. Lanes 1 and 4, coexpression of SCA3trQ78 and control transgene (*UAS-eGFP*); lanes 2 and 5, coexpression of SCA3trQ78 and Atx2; lanes 3 and 6, coexpression of SCA3trQ78 and Atx2ΔP (genotypes in [A], [E], [G]). At 24 h, only flies coexpressing Atx2, but not Atx2ΔP, have high-molecular weight complexes containing Atx3 visible in the stacking gel portion (s) of the blot.

The formation of nuclear inclusions containing pathogenic polyglutamine protein is a hallmark of polyglutamine diseases, with the size and number of inclusions typically correlating with disease severity in animal models [1–3]. In retinal sections of animals expressing pathogenic Atx3 driven by *Rh1-Gal4*, inclusions were sparse at 24 h but prominent by 4 d (Figure 4A and 4B). Up-regulation of Atx2—which enhances Atx3 toxicity (Figures 1 and 2)—also accelerated inclusion formation, such that at 24 h, Atx3-positive inclusions were now prominent (Figure 4E). By 4 d, the inclusions were similar to those of animals expressing pathogenic Atx3 alone (compare Figure 4F to 4B).

Immunoblot experiments showed similar results (Figure 4J). At the 24-h time point, coexpression of normal Atx2 with pathogenic Atx3 caused the early appearance of SDS-insoluble Atx3 complexes that remain within the stacking gel (compare lanes 1 and 2), presumably reflecting protein accumulations that correlate with the early appearance of nuclear inclusions in cryosections. At the latter 4-d time point, insoluble Atx3 complexes were present in extracts from both groups (lanes 4 and 5). Differences in the inclusions and high molecular weight complexes were due to post-transcriptional causes, since Atx2 coexpression did not affect the transcript levels of the SCA3trQ78 transgene (Figure 4I).

Endogenous Atx2 protein was normally cytoplasmic throughout the retina. By 4 d, however, endogenous Atx2 was detectable in most Atx3-positive nuclear inclusions (Figures 4B–4D and S3), reminiscent of the Atx2 localization to inclusions in SCA3 patients [7]. Up-regulated Atx2 protein was not selectively recruited to inclusions, but remained punctate along the length of photoreceptor neurons; this pattern did not change over time or with coexpression of SCA3trQ78 (Figure S4). Notably, in both SCA2 patients and a SCA2 mouse model, pathogenic Atx2 with expanded polyglutamine is present in a similar cytoplasmic, punctate pattern [33]. Whereas inclusions of pathogenic Atx3 co-label for chaperones such as Hsp70, no such inclusions were observed in flies expressing only high levels of Atx2 (Figure S4). Western blot analysis confirmed that Atx2 protein levels were not affected by pathogenic Atx3 (unpublished data). In summary, endogenous Atx2 is recruited to nuclear inclusions formed by pathogenic Atx3, as in SCA3 patients.

### The Interaction between Atx2 and Pathogenic Atx3 Is Dependent on the PAM2 Motif of Atx2

**Figure 5:**
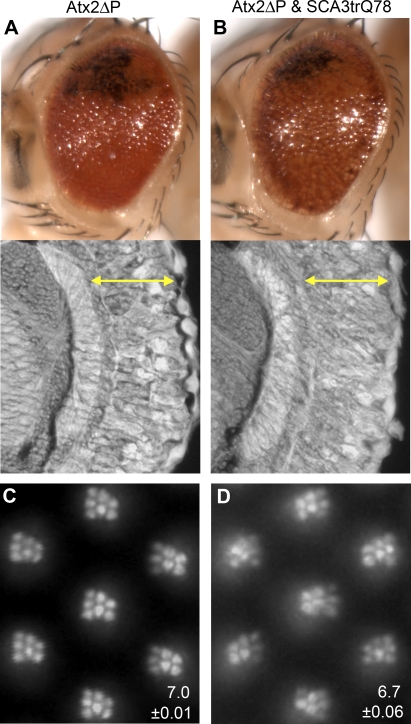
The PAM2 Motif of Atx2 Is Required for Enhancement of Atx3 Degeneration and for Atx2 Toxicity (A and B) External eye and internal retinal sections. Retinal thickness is indicated by double-headed yellow arrows. (A) Expression of Atx2 lacking the PAM2 motif (Atx2ΔP) causes a disrupted eye with (top) external roughness and dorsal discoloration and (below) disorganized internal structure. Genotype: *Gmr-Gal4/+; UAS-Atx2ΔP/+*. (B) Co-expression of Atx2ΔP with SCA3trQ78 does not enhance SCA3trQ78 degeneration; the eye looks identical to that of flies expressing Atx2ΔP only. Genotype: *Gmr-Gal4/+; UAS-SCA3trQ78(w)/UAS-Atx2ΔP*. (C and D) Pseudopupil preparations to highlight internal retinal degeneration. (C) Flies expressing Atx2ΔP maintain a normal retina over time, with a mean of seven PR (18 d shown, genotype: *Rh1-Gal4/UAS-Atx2ΔP*). (D) Coexpression of Atx2ΔP with SCA3trQ78 has minimal effect on SCA3trQ78 degeneration, with a mean of 6.7 PR (6 d, *UAS-SCA3trQ78(s)/+; Rh1-Gal4/UAS-Atx2ΔP*); normally SCA3trQ78 expression by itself results in a mean of 6.9 PR at 6 d (see Figure 2A).

To provide insight into the mechanism by which Atx2 modulates polyQ toxicity, we determined whether the conserved 12-amino acid PAM2 motif of the Atx2 protein was required for enhancement of SCA3 toxicity. The PAM2 motif is required for Atx2 to associate with PABP, and together with the RNA-binding Lsm domain, it mediates the colocalization of Atx2 with PABP in polyribosomes [16]. We tested whether a construct lacking the conserved PAM2 domain, *UAS-Atx2ΔP* [16], could modulate Atx3 toxicity. Atx2ΔP retains partial Atx2 function, since it delays embryonic lethality caused by complete loss of *atx2* function [16]. The Atx2ΔP transgenic line expressed the protein at a level comparable to that of the strongly expressing Atx2(s) line (Figure S5). Expression of Atx2ΔP driven by *Gmr-Gal4* caused a disrupted eye phenotype distinct from that caused by up-regulation of normal Atx2: black patches were present on the surface, and the underlying retina was disorganized although intact, showing no evidence of degeneration (Figure 5A). In striking contrast to normal Atx2 protein, coexpression of Atx2ΔP failed to enhance SCA3trQ78 toxicity: the flies showed an eye phenotype identical to that caused by Atx2ΔP alone (Figure 5B; compare to Figure 1D).

We confirmed this finding by examining interactions between Atx2ΔP and pathogenic Atx3 in the adult, uncomplicated by any developmental effects. Coexpression of Atx2ΔP and SCA3trQ78 driven by *Rh1-Gal4* caused a mild adult-onset photoreceptor loss at 6 d (6.7 ± 0.06 PR; Figure 5D) that was similar to that caused by SCA3trQ78 alone (6.9 ± 0.02 PR; see Figure 2A), and strikingly less severe than that caused by coexpression of SCA3trQ78 with normal Atx2 (*p* < 0.001; see Figure 2C). The accelerated appearance of inclusions caused by up-regulated normal Atx2 was also dependent on the PAM2 domain, since coexpression of Atx2ΔP with SCA3trQ78 did not alter the time course of inclusion formation (compare Figure 4G to 4E). Moreover, coexpression of Atx2ΔP did not cause the early appearance of SDS-insoluble complexes containing Atx3 (Figure 4J, compare lane 3 to lane 2). Taken together, these data show that the PAM2 motif of Atx2 is required for the enhancement of pathogenic Atx3-dependent degeneration as well as for the early appearance of Atx3 inclusions and insoluble complexes.

Adult-onset expression of Atx2 lacking the PAM2 motif alone also did not cause neurodegeneration: even at 18 d, flies expressing Atx2ΔP had a normal retinal structure (7.0 ± 0.01 PR, Figure 5C; compare to normal Atx2 expressed at an equivalent level, which results in 1.5 ± 0.08 PR, Figure 2H). Thus the PAM2 motif is required for the inherent toxicity of Atx2 when it is up-regulated in the adult eye.

### The Atx2-Binding Protein PABP Modulates Neurodegeneration

**Figure 6:**
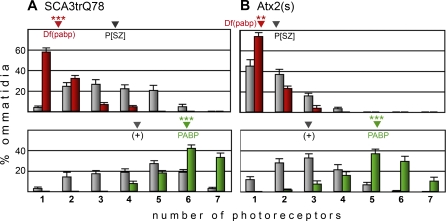
The Atx2 Binding Partner PABP Modulates Neurodegeneration Distributions of ommatidia from pseudopupil analysis; mean PR counts indicated by the positions of triangles above the graphs (see text for precise values). (A) Flies expressing pathogenic Atx3 at 18d. Top: red bars show distribution of ommatidia in flies heterozygous for a deletion removing *pabp* (Df(pabp)) compared to flies heterozygous for a P element used to synthesize the deletion (grey; P[SZ]). The loss of 50% of PABP activity (red) results in significant enhancement of degeneration (*** *p* < 10^−4^). Genotypes: *UAS-SCA3trQ78(s)/*P[5-SZ-3325]*; Rh1-Gal4/+* or *UAS-SCA3trQ78(s)/*Df(2R)ED3610*; Rh1-Gal4/+*. Bottom: up-regulation of PABP (green) suppresses degeneration induced by SCA3trQ78 (*** *p* < 10^−4^). Genotypes: *UAS-SCA3trQ78(s)/+; Rh1-Gal4/+* or *UAS-SCA3trQ78(s)/+; Rh1-Gal4/UAS-PABP*. (B) Strong expression of Atx2 in 6-d flies. Top, degeneration by Atx2 (grey) is enhanced by reduced levels of PABP (red; ** *p* = 0.003). Bottom, up-regulation of PABP (green) suppresses Atx2-mediated degeneration (*** *p* < 10^−4^). Genotypes as in (A), except with *UAS-Atx2(s)* instead of *UAS-SCA3trQ78*.

PABP is the only known protein to date that interacts directly with Atx2 through the PAM2 motif [13,14,16]; therefore, given the important role of the PAM2 motif described above, we asked if PABP played a role in SCA3 neurodegeneration. Heterozygosity for the available *pabp* allele had no effect on Atx3 toxicity, although this allele is unlikely to be a complete loss of function [34]. We then tested a deletion chromosome that removed the *pabp* gene, comparing to appropriate control lines (see Materials and Methods). Flies expressing pathogenic Atx3 that were heterozygous for this deletion showed dramatically enhanced photoreceptor loss (1.6 ± 0.07 PR, compared to the control 3.5 ± 0.16 PR; Figure 6A). Control experiments confirmed that the deletion alone, in the absence of pathogenic Atx3, did not cause neurodegeneration (unpublished data). In contrast to the loss-of-function situation, overexpression of PABP significantly suppressed neurodegeneration (from 4.2 ± 0.18 PR to 6.0 ± 0.08 PR; Figure 6A). These observations indicated that PABP has the opposite activity as Atx2 with respect to Atx3-dependent neurodegeneration: whereas Atx2 enhances the toxicity of Atx3, PABP is protective.

We then tested whether PABP could modulate the degeneration induced by strong expression of Atx2 as shown in Figure 2D and 2H. Decreased PABP function enhanced Atx2-dependent photoreceptor loss (from 1.7 ± 0.09 PR to 1.3 ± 0.06 PR); likewise, up-regulation of PABP protected against photoreceptor degeneration (2.9 ± 0.16 PR to 5.1 ± 0.19 PR; Figure 6B). These studies suggest that the toxicity of Atx2 is mitigated by physical association with PABP, and they are consistent with PABP also playing a crucial role in the Atx2–Atx3 interaction. Together with our results demonstrating the crucial role of the PAM2 motif, these data highlight the importance of the normal biological activity of Atx2 and of PABP in modulating the toxicity of pathogenic Atx3.

## Discussion

Our studies in *Drosophila* reveal that the toxicity of pathogenic human Atx3 is critically dependent on Atx2 activity. Reduction of endogenous Atx2 function mitigated Atx3-induced neurodegeneration, and up-regulation of Atx2 synergistically enhanced degeneration. We also reveal the roles in neural integrity played by the non-polyglutamine PAM2 motif of Atx2 and by PABP, which binds to Atx2 via this motif. These data are consistent with and expand upon clinical findings suggesting interactions between Atx2 and Atx3 in human disease [5–7]. In the fly, endogenous Atx2 colocalized with pathogenic Atx3 in inclusions, as seen in human patients [7], with up-regulation of Atx2 enhancing Atx3 toxicity concomitant with a faster onset of inclusions and of SDS-insoluble complexes. These findings suggest that therapeutic approaches to modulate Atx2 activity may be effective against multiple disease situations, including SCA2 and SCA3.

Interestingly, we find that normal Atx2 is toxic, causing degeneration when up-regulated. Previous animal models have demonstrated that normal protein products associated with SCA1 and Parkinson's disease—Ataxin-1 and α-Synuclein, respectively—are also toxic when expressed at sufficiently high levels [9,29,30]. Expansion of the polyglutamine domain in Ataxin-1 or Parkinson disease-associated missense mutations of α-Synuclein presumably lead to increased levels of the respective proteins, sufficiently high to elicit disease. Up-regulation of *Drosophila* Atx2 may cause degeneration for similar reasons. Our studies further reveal that neuronal toxicity of Atx2 depends on its PAM2 motif—an observation with an interesting parallel to Ataxin-1, the protein that causes SCA1: an expanded polyglutamine repeat in Ataxin-1 is not sufficient to cause neurodegeneration in mouse models for SCA1, but rather pathogenic Ataxin-1 also requires its AXH domain to cause disease [35].

The importance of the PAM2 motif for Atx2′s toxicity and for the enhancement of Atx3 toxicity suggests a clue to the mechanism of the interaction. The PAM2 motif has been shown to bind specifically to the PABC domain [13], with PABP being currently the only known PABC-containing protein that interacts with Atx2. PABP is a ubiquitously expressed and essential protein that binds to the polyadenylated tails of mRNAs and is required for their translation. Furthermore, biochemical and genetic data support an interaction between Atx2 and PABP across many species [15,16,36]. Data from C. elegans indicate that loss of Atx2 can result in misregulated translation [15], and in yeast Atx2 negatively regulates PABP [36]. Consistent with these findings, we have shown that Atx2 and PABP have opposing activities in modulating the progression of SCA3 toxicity in flies.

Protein interaction studies indicate that Atx2 and Atx3 do not interact directly; in a survey of the interaction network of ataxia-associated proteins, Atx2 and Atx3 were separated by four nodes [12]. However, the known function of PABP and the role of the PAM2 motif in localizing Atx2 to polyribosomes [16] together indicate that Atx2 and PABP modulate translation of specific transcripts. Since Atx2 is sufficient to cause neurodegeneration in the absence of pathogenic Atx3 (see Figure 2D and 2H), Atx3 mRNAs cannot be the sole target of Atx2–PABP interactions, and additional transcript targets must be critical to normal neuronal integrity. 

Our experiments in *Drosophila* demonstrate that the fly provides an outstanding complement to clinical observations and to vertebrate disease models. In this case, the fly has highlighted the significance of intriguing interactions between the genes that cause SCA2 and SCA3 diseases that can be supported by molecular and genetic findings. More specifically, these data indicate striking crosstalk between the pathways of normal Atx2 function and pathogenic Atx3 activity. Further understanding of both the Atx2 and Atx3 pathways may reveal insight into maintenance of neuronal integrity in a number of distinct disease situations.

## Materials and Methods

### Fly stocks.

Truncated and full-length *UAS-SCA3* transgenes have been previously described [8,21]. The following stocks were kind gifts from Terry Satterfield and Leo Pallanck: *UAS-Atx2* (a.k.a. *UAS-Atx2.1B*), *UAS-Atx2(s)* (a.k.a. *UAS-Atx2.4*), *UAS-Atx2ΔP* (a.k.a. *UAS-Atx2ΔC1A3*), and FRT82B *atx2* ^X1^. *UAS-Htt-ex1Q93* [24] was a gift from Larry Marsh, Leslie Thompson (University of California Irvine), and Larry Goldstein (University of California San Diego), and is termed Htt174(Q93) in Figure S1; *UAS-HttQ128* [25] was a gift of Troy Littleton (MIT), and is termed Htt676(Q128). MARCM stocks (see below) and *UAS-PABP* [34] were obtained from the Bloomington Stock Center. EP(3)3145, Df(2R)ED3610, P[5-SZ-3325], P[CB-6101–3], Df(3R)ED5705, P[5-SZ-3416], and P[CB-0741–3] were obtained from the Szeged Stock Center. P[CB-6101–3] and P[5-SZ-3325] were P element insertions used to construct Df(2R)ED3610, which deletes *pabp*, and P[5-SZ-3416] and P[CB-0741–3] were used to construct Df(3R)ED5705, which deletes *atx2* [37]. Both P insertions of a pair were equivalent controls for comparisons to the respective deletions in the experiments; results are shown for P[5-SZ-3325] only in Figure 6 for simplicity. All experiments were performed at 25 °C. The EP(3)3145 allele of *atx2* was identified in the part of the screen described in [38] where flies bearing *Gmr-Gal4* and *UAS-SCA3trQ78* were crossed to a series of enhancer P (EP) insertions that confer Gal4-dependent expression on adjacent genes [39].

### External eye microscopy.

For eye pictures in Figures 1 and 5, we used a Leica Z16-Apo A motorized zoom microscope system with DFC420 digital camera and Leica Application Suite Montage module software (Leica Microsystems).

### Pseudopupil preparations.

Pseudopupil preparations were performed as described [40] with *w* ^+^ male flies. The *Rh1-Gal4* driver is active in the outer six PR neurons of each unit eye (ommatidium). As an indicator of the number of PR neurons, rhabdomeres were counted in 10–20 ommatidia of *n* = 10 flies of each genotype. Mean PR counts ± standard error of the mean (SEM) per ommatidium are indicated in the figures and text. Statistical analysis was performed with Prism software. The two-tailed Mann Whitney test was used to calculate the *p*-value when comparing the results of pseudopupil preparations between two groups. In experiments with three groups (in Figures 2I, 2J, and S2, and for a comparison of phenotypes due to SCA3trQ78 alone, with Atx2 or with Atx2ΔP, stated in the text discussing Figure 5D), the Kruskal-Wallis test was performed followed by Dunn post-tests.

### Quantitative real-time PCR.

Quantitative real-time PCR was performed in triplicate on each cDNA sample with an Applied Biosystems 7500 Fast system. Duplex PCRs were performed in single wells with human *ATXN3* forward primer 5′CAGGACAGAGTTCACATCCATGT, reverse primer 5′GCCTTACCTAGATCACTCCCAAGT, and FAM-labeled Taqman probe 5′CCAGCCACCAGTTCAG (Applied Biosystems assay by design), and with human 18s rRNA as the internal control (the *Drosophila* sequence is identical; primers and VIC-labeled probe, Applied Biosystems #4319413E). Template was random-primed cDNA prepared with Superscript III (Invitrogen) from RNA made from 80–115 male heads (Qiagen RNeasy). Before PCR, cDNA was treated with Turbo DNase (Ambion) to remove genomic DNA. Quantitative PCR results were analyzed with the two-tailed *t*-test (Prism software).

### Immunoblots.

Heads were extracted in Laemmli buffer and samples were run on 12% polyacrylamide gels and blotted overnight at 20 V at 4 °C (all reagents from Bio-Rad). Antibody dilutions and block were 3% dry milk in Tris-buffered saline with Tween (Sigma). Imaging was performed with ECL+ (GE Healthcare) on a Fuji LAS-3000 system. Antibodies: rat anti-HA-peroxidase (Roche; 1:500), rabbit anti-Atx2 [22] (1:10,000), mouse anti-tubulin (Developmental Studies Hybridoma Bank; 1:2,000), mouse anti-β-actin (Abcam; 1:20,000) with secondaries anti-rabbit-peroxidase and anti-mouse-peroxidase (Chemicon; 1:2,000).

### Marked wing margin clones.

Marked wing margin clones were induced using previously developed reagents and procedures [32]. MARCM stock females were crossed to experimental FRT82B males and allowed to lay eggs for approximately 18 h, at which point parental flies were cleared from the vials. Progeny were of genotype *Elav-Gal4 hsFLP UAS-mcd8GFP*/Y; [*UAS-SCA3trQ78(s)*]/+; FRT82B *tubP-Gal80*/FRT82B [*atx2^X1^*]. The presence of *UAS-SCA3trQ78* or *atx2* ^X1^ varied between experimental groups, as indicated by brackets. Mitotic recombination between the FRT sites was induced in progeny larvae 3 d later by submerging vials for 50 min in a 37 °C water bath. About half of the resulting daughter cells lacked Gal80 (a Gal4 inhibitor), and if they or their progeny were neurons, the neuronal driver *Elav-Gal4* was free to drive expression of the GFP marker and (if present) of SCA3trQ78 as well. If the *atx2* ^X1^ mutation was present on the FRT82B chromosome as indicated above, these daughter cells and their progeny were homozygous for this null *atx2* allele.

### Histology.

Flies in Figures 1, 5, and S1 were 1d *w* ^1118^ males. Retinal tissue was visualized by autofluorescence in horizontal paraffin sections [29]. Antibody staining was performed on frontal cryosections as described [40]. The SCA3trQ78 transgene encodes a protein tagged with the HA epitope [8]. Antibodies used were mouse anti-HA (5B1D10, Zymed, 1:100); rabbit anti-Atx2 [22] (1:5,000); and mouse anti-hsc70/hsp70 (SPA-822, Stressgen, 1:100). Secondary antibodies used were anti-mouse-alexa488, anti-mouse-alexa594, and anti-rabbit-alexa594 (1:100; Invitrogen-Molecular Probes).

## Supporting Information

Figure S1 Atx2 Enhances Toxicity of the Exon-1 Model of Huntington Disease Two *Drosophila* models for Huntington disease comprise different N-terminal portions of human Huntingtin, a protein of over 3,000 amino acids. One transgene expresses the first exon of the Htt gene, comprising the N-terminal 174 residues including a polyQ expansion of Q93, termed here Htt174(Q93) [24]. The second expresses the first 676 residues of Htt including a repeat expansion of Q128, Htt676(Q128) [25].
(A–D) External eye and internal retinal sections. Double-headed yellow arrows indicate retinal depth, which highlights internal degeneration. (A) Expression of Htt174(Q93) caused internal retinal degeneration (genotype *Gmr-Gal4/UAS-Htt-ex1Q93*) that (B) was enhanced by Atx2 up-regulation (*Gmr-Gal4 UAS-Atx2/UAS-Htt-ex1Q93*) (compare to control in Figure 1A and to Figure 1C, showing the mild developmental defect of Atx2 alone). (C) Expression of Htt676(Q128) (*Gmr-Gal4/UAS-HttQ128*) caused degeneration that was not changed upon (D) up-regulation of Atx2 (*Gmr-Gal4 UAS-Atx2/UAS-HttQ128*).
(5.2 MB TIF)

Figure S2 Degeneration due to Full-Length Pathogenic Atx3 Is Partially Suppressed by the Loss of 50% of Atx2 Activity Adult-onset degeneration analyzed by pseudopupil method showing the distribution of unit eyes or ommatidia at 43–45 d. Df(3R)ED5705 is a deletion that removes *atx2*; P[5-SZ-3416] and P[CB-0741–3] are isogenic P element insertion lines that were used to synthesize the deletion [37]. Mean numbers of PR ± SEM per ommatidium for *n* = 10 flies, indicated schematically at top by arrowheads at the appropriate place along the *x*-axis, are as follows: P[5-SZ-3416] control, in black: 3.0 ± 0.13; P[CB-0741–3] control, in grey: 3.5 ± 0.15; Df(3R)ED5705, in green: 5.0 ± 0.13 (** *p* < 0.001 vs. P[5-SZ-3416] and *p* < 0.01 vs. P[CB-0741–3]). Genotypes: black, P[5-SZ-3416]*/Rh1-Gal4 UAS-SCA3Q78*; grey, P[CB-0741–3]*/Rh1-Gal4 UAS-SCA3Q78*; and green, Df(3R)ED5705*/Rh1-Gal4 UAS-SCA3Q78*.
(225 KB TIF)

Figure S3 Atx2-Atx3 Colocalization Larger regions of the same sections shown in Figure 4B–4D of the main text, with *Rh1-Gal4* driving expression of pathogenic Atx3 only. DAPI stain (top) highlights the orientation and nuclear localization of inclusions containing pathogenic Atx3 (red, anti-HA) and endogenous Atx2 (green, anti-Atx2).
L, nuclei of the lamina, the brain structure to which most photoreceptors synapse; R, retinal nuclei, including those of photoreceptor neurons R1–R7, are concentrated at the outer edge of the retina; R8, nuclei of photoreceptors R8, which are not visible in pseudopupil preps.
(6.0 MB TIF)

Figure S4 Distribution of Up-Regulated Atx2 in Retinal Cryosections Males with *Rh1-Gal4* driving the following UAS transgenes: (A) Atx2 only, (B–G) Atx2 and SCAtrQ78(s), (H–J) SCA3trQ78(s) only, (K–M) Atx2(s) only. (A, B, E, and K) Anti-Atx2 in green; (C and F) anti-HA detecting pathogenic Atx3 in magenta; (I and L) anti-hsc70/hsp70 in magenta. (D, G, J, and M) merged green and magenta images. (A, B, and E) The pattern of up-regulated Atx2 is similar over time and with coexpression of SCAtrQ78(s). (D and G) Up-regulated Atx2 colocalizes with pathogenic Atx3, but is also widely expressed throughout the depth of the retina. (H–J) An endogenous chaperone strongly colocalizes with pathogenic Atx3 in inclusions, but (K–M) is more uniformly diffuse in the presence of strong Atx2 up-regulation.
(6.7 MB TIF)

Figure S5 Expression Levels of Atx2 Transgenes Each lane contains an extract of heads from 1-d males, bearing either the *Gmr-Gal4* or the *Rh1-Gal4* driver and the indicated UAS transgene. Endogenous levels of Atx2 (lane Rh1>eGFP) were not consistently detectable. Asterisks indicate nonspecific bands. Quantification of Atx2 transgene levels of Rh1-Gal4–dependent expression indicated that the Atx2(s) and Atx2Δ*P* insertions expressed at levels 2.3 ± 0.6-fold and 2.5 ± 0.6-fold stronger than Atx2, respectively (*n* = 3 independent experiments, quantification with ImageJ software). Atx2 expression resulting from *Gmr-Gal4* is higher than from *Rh1-Gal4*, since *Gmr-Gal4* is active in most cells in the developing eye tissue, whereas *Rh1-Gal4* is expressed in only six photoreceptor neurons of each ommatidium.
(10.1 MB TIF)

### Accession Numbers

The Online Mendelian Inheritance in Man (http://www.ncbi.nlm.nih.gov/sites/entrez?db=omim) accession numbers for the genes and gene products mentioned in this paper are as follows: Atx3 (607047), SCA2 (183090), and SCA3 (109150).

The FlyBase (http://flybase.bio.indiana.edu) accession numbers for the genes and gene products mentioned in this paper are as follow: *atx2* (FBgn0041188) and PABP (FBgn0003031).

## Abbreviations

Atx: Ataxin
GFP: green fluorescent protein
Htt: Huntingtin
PABP: poly(A)-binding protein
PR: photoreceptor
SCA: spinal cerebellar ataxia
SEM: standard error of the mean

## Note Added in Proof

While this manuscript was under review, Al-Ramahi et al [41] reported similar activity of Atx2 on a fly SCA1 model that further supports the idea of interactions among SCA genes.
